# Inflammatory markers and the risk of idiopathic sudden sensorineural hearing loss: A Mendelian randomization study

**DOI:** 10.3389/fneur.2023.1111255

**Published:** 2023-02-22

**Authors:** Tingfeng Zhou, Mengjiao Chen, Ziyi Yuan, Zhigang Xia, Shurou Zhang, Ziheng Zhang, Huanqi Chen, Renyu Lin

**Affiliations:** ^1^Department of Otolaryngology, First Affiliated Hospital of Wenzhou Medical University, Wenzhou, China; ^2^Wenzhou Medical University, Wenzhou, China

**Keywords:** C-reactive protein, inflammation, TNF-α, fibrinogen, idiopathic sudden sensorineural hearing loss, Mendelian randomization

## Abstract

**Background:**

Observational studies suggest that inflammatory markers may increase the risk of idiopathic sudden sensorineural hearing loss (ISSHL). However, the causal relationship between the two has not been established. We sought to assess the possible causal effect between several genetically predicted inflammatory markers and ISSHL by Mendelian random (MR) analysis.

**Methods:**

We extracted single nucleotide polymorphisms (SNPs) associated with C-reactive protein (CRP), Tumor necrosis factor-α (TNF-α), and fibrinogen from abstract data from the European Individual Large genome-wide association studies (GWAS). Genetic data for ISSHL were obtained from the FinnGen study (*n* = 196,592). Effect estimates were assessed using inverse variance weighting (IVW) as the primary method. Sensitivity analyses were performed using weighted median, MR-Egger, and MR-PRESSO to evaluate heterogeneity and pleiotropy.

**Results:**

In the random-effects IVW approach, there was a significant causal relationship between genetic susceptibility to CRP levels and ISSHL (OR = 1.23, 95% CI = 1.02–1.49, *P* = 0.03). In contrast, genetic TNF-α and fibrinogen were not risked factors for ISSHL (OR = 1.14, 95% CI = 0.88–1.49, *P* = 0.30; OR = 0.74, 95% CI = 0.07–7.96, *P* = 0.30; OR = 1.05, 95% CI = 0.88–1.25, *P* = 0.59). All the above results were consistent after validation by different Mendelian randomization methods and sensitivity analyses.

**Conclusion:**

This Mendelian randomization study provides causal evidence that CRP is a risk factor for ISSHL, while TNF-α and fibrinogen do not increase the risk for ISSHL Introduction.

## 1. Introduction

Idiopathic sudden sensorineural hearing loss (ISSHL) is a typical otolaryngological emergency of unknown etiology, defined as a sensorineural hearing loss of at least 30 dB at least three consecutive frequencies for at least 72 h, which may be accompanied by tinnitus, a sensation of ear congestion and vertigo (1). According to the World Health Organization (WHO), an estimated 2.5 billion people worldwide will suffer varying degrees of hearing loss by 2050 (2). At the same time, it is the most common cause of disability worldwide and the third most common cause of lost productivity for several years due to disability, which severely affects patients' quality of life (3). Therefore, continued research into the underlying complex pathophysiological mechanisms can provide practical prevention and treatment strategies for patients with ISSHL in the clinical setting.

Previous studies have shown that inflammatory factors may play a crucial role in developing ISSHL by increasing the risk of microvascular injury and ischemia during these processes (4). Previous studies have found a possible significant association between C-reactive protein (CRP), a representative biomarker of systemic inflammation, and ISSHL (5, 6). Another inflammatory marker, tumor necrosis factor-α (TNF-α), is an immunomodulatory factor that is also thought to influence the development of ISSHL (7, 8). Fibrinogen, an essential indicator of blood viscosity, may also be involved in the development and prognosis of ISSHL (9). However, these findings have been refuted in other studies (10). A recently published meta-analysis has not clarified their association with ISSHL (11). These contradictory results may result from potential confounders and reverse causality in traditional observational studies, so the causal relationship between them remains an open question.

Mendelian randomization (MR) is an emerging genetic epidemiological approach based on the principle of using genetic variation as an instrumental variable (IV) for risk factors, thereby fully elucidating the causal effect of exposure on outcome (12). Since genes are randomly assigned in meiosis, this approach eliminates other potential confounders and interferences through reverse causality, leading to more meaningful causal conclusions than conventional observational studies (13). Therefore, in this study, we performed a two-sample MR analysis to clarify further the causal hypothesis of CRP, TNF-α, and fibrinogen and their association with ISSHL risk using existing, publicly available, large-scale genome-wide association studies (GWAS).

## 2. Methods

### 2.1. Study design

**Figure 1:**
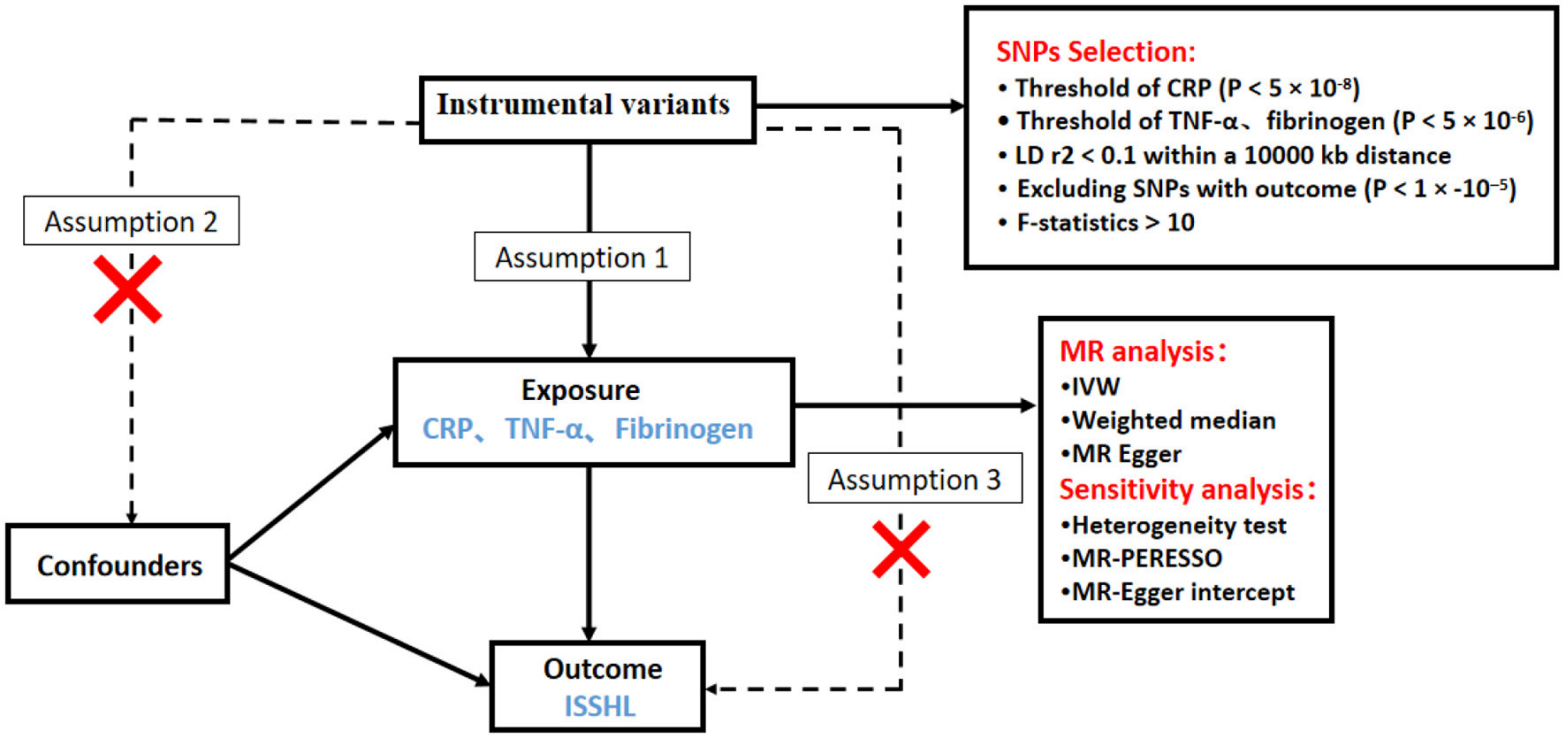
Schematic diagram of the Mendelian randomization study design process of Inflammatory markers and ISSHL.

We investigated the causal relationship between inflammatory markers and ISSHL using a two-sample MR design. A convincing MR design should be based on three key assumptions: (1) genetic variation is directly and strongly associated with exposure (inflammatory markers); (2) genetic variation is not associated with potential confounders, and (3) genetic variation affects outcome (ISSHL) only through exposure and not through other pathways (14). Figure 1 shows an overview of this MR approach with two samples. All summary data used in this study are publicly available and restricted to European populations, and appropriate ethical approval and informed patient consent were obtained from participants for all previous studies. This paper is published by the recommendations of the Statement on Strengthening the Reporting of Observational Studies in Epidemiology Using Mendelian Randomization (STROBE-MR) (15).

### 2.2. GWAS data for inflammation markers

The IV for CRP was derived from the most extensive available meta-analysis of European individual genome-wide association studies (GWAS). The cohort study drew on 88 previous pooled statistics comprising 204,402 individuals (16). Ligthart et al. relied on standard laboratory techniques to measure serum CRP concentration and excluded individual and genetic variation during the analysis according to quality control criteria. Further details on participant characteristics, genotype data, and statistical models are described in the published GWAS. In addition, the genetic toolkit for fibrinogen was derived from GWAS collected from 3301 European subjects in the Sardinia project (17). For genetic variation in TNF-α, the authors recruited 3,454 people from FinnGen (18).

### 2.3. GWAS data for ISSHL

Genetic information related to ISSHL was obtained from publicly available GWAS abstract summary data, which are accessible through the GWAS catalog (https://gwas.mrcieu.ac.uk/). This study's most recently published GWAS included 196,592 participants (1,491 cases and 195,101 controls).

### 2.4. Instrumental variable selection

A criterion for genetic instrument selection was developed to select scientifically valid SNPs. When CRP was used as the exposure, SNPs (*p* < 5 × 10^−8^) were chosen as instrumental variables to achieve genome-wide significance. However, given the limited sample size and number of SNPs, we loosened the association threshold with *p* < 5 × 10^−6^ when selecting SNPs associated with TNF-α and fibrinogen. To avoid the effects of linkage disequilibrium, *r*^2^ < 0.1 at a distance of 10,000 kb (19). To meet the third central assumption (that IV variants affect outcome only by exposure), SNPs directly associated with hearing loss outcome (*p* < 1 × 10^−5^) were also removed in each analysis. Finally, we calculated the overall F-statistic: *F* = $\frac{beta^{2}}{se^{2}}$ (20). In this process, we selected SNPs with *F* > 10 to ensure that each SNP had sufficient strength for the analysis. Summary data on single nucleotide polymorphisms associated with exposure and their association with ISSHL can be found in Supplementary material 1.

### 2.5. Statistical analyses

We performed random Mendelian effects analyses to assess the causal effects between CRP, TNF-α, fibrinogen, and ISSHL.

First, we regressed genetic variance in exposure (CRP, TNF-α, fibrinogen) on the outcome (genetic variance in susceptibility to ISSHL), with each conflict representing one data point, and analyzed the causal relationship between exposure and ISSHL using an inverse variance-weighted random effects (IVW) method with a *P* < 0.05 in the principal analysis. This method produces estimates expected to exceed the Wald ratio estimates of variance, so we chose it as the primary method for this MR (21).

In addition, we performed several sensitivity analyses using MR-Egger, weighted medians, and outliers (MR-Presso) as a complement to IVW to identify any bias in evaluating MR hypotheses. MR-Egger analyses allow for pleiotropy for all genetic variants, but the magnitude of pleiotropy (from genetic variant to outcome, bypassing exposure) should be separate from the main effect (from genetic variation to exposure) extent. Apart from this, we also applied the MR-Egger intercept test to detect unbalanced horizontal pleiotropy. If pleiotropy was present, then the analysis yielded a *p* < 0.05 for the intercept (22). The weighted median provides a robust estimate even if up to 50% of the genetic variation violates the assumption (23). The MR-PRESSO method can also detect and remove outliers to obtain relatively unbiased estimates (24). For the meaningful forecast, we used Cochrane *Q*-values to assess heterogeneity and visualized funnel plots by plotting the inverse distribution of standard errors for each SNP around the MR estimates (22). In the leave-one-out method, each SNP was removed in turn, and the remaining SNPs were used to calculate the causal effect of gene prediction exposure (CRP, TNF-α, fibrinogen) on the outcome (ISSHL) (25).

All MR analyses in this study were performed using R software (version 4.2.1) and the “TwoSampleMR” package (version 0.5.6).

### 2.6. Causal association of CRP with ISSHL

**Figure 2:**
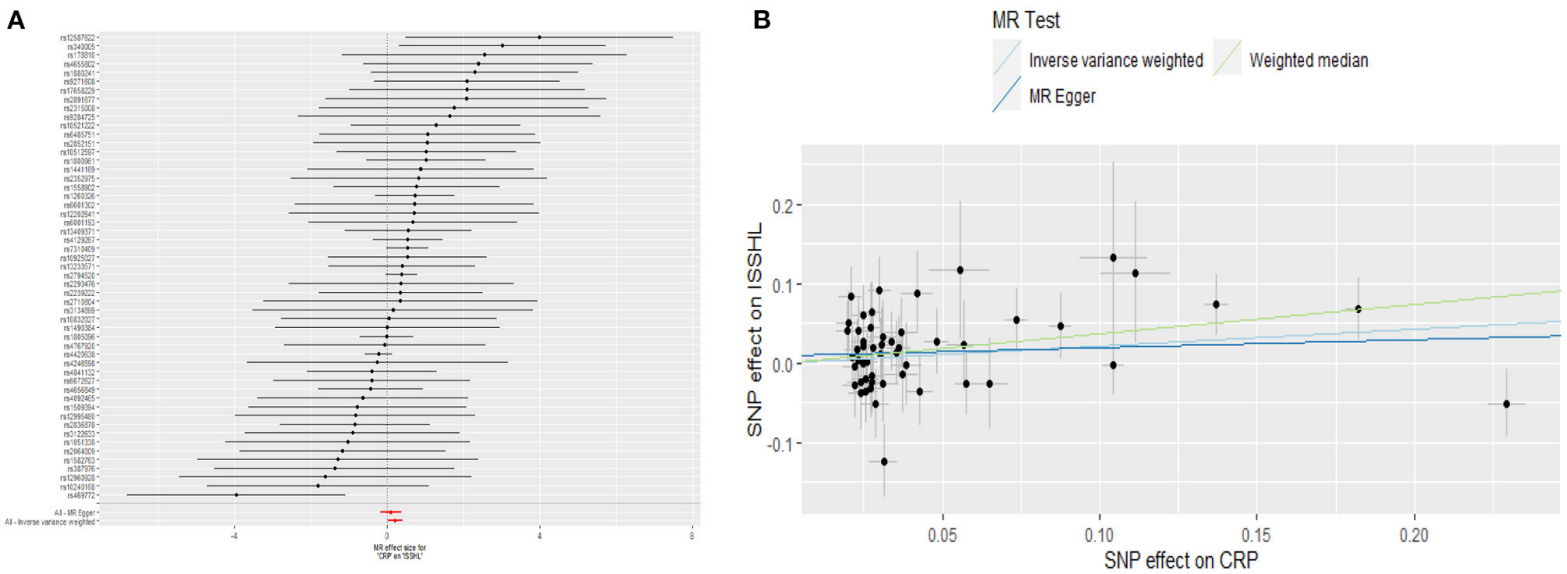
**(A)** Forest plot and **(B)** scatter plot of the potential effects of CRP associated SNPs on ISSHL.

**Figure 3:**
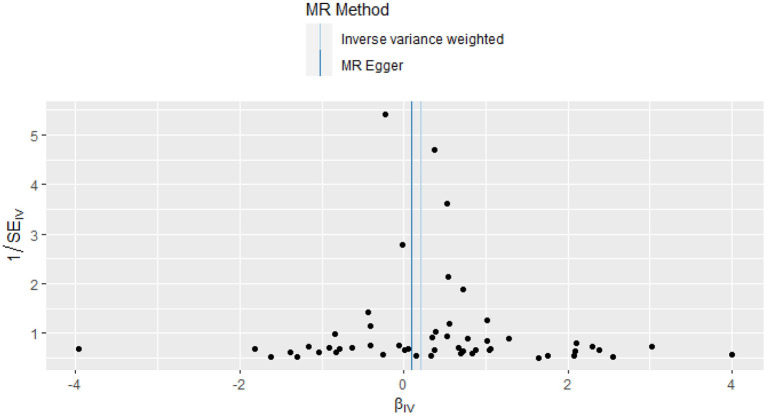
Funnel plot of the causal effect of CRP related SNPs on ISSHL.

**Figure 4:**
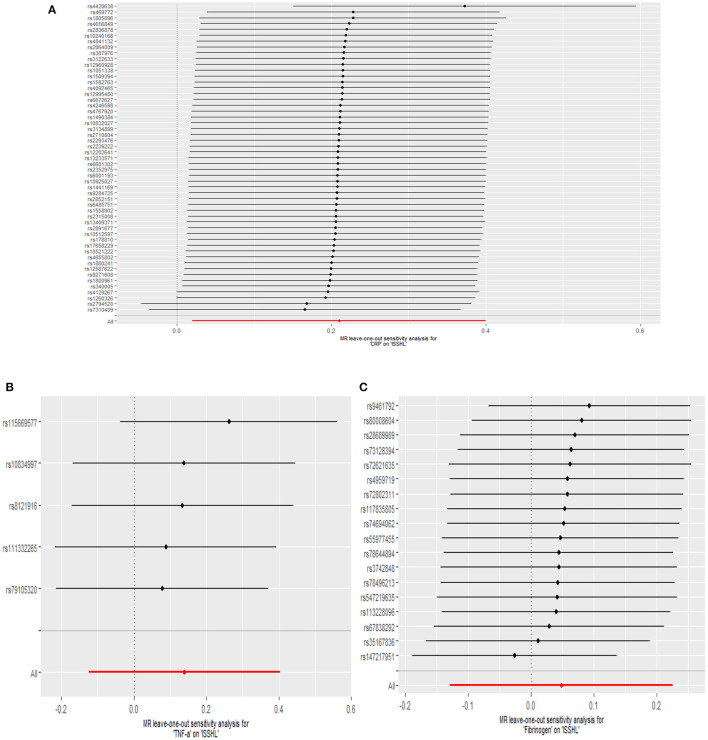
Leave-one-out plots for the MR analyses of ISSHL on CRP **(A)**, TNF-α **(B)**, and Fibrinogen **(C)**.

This study investigated the causal effect of CRP levels and ISSHL using MR with two samples. The detailed results can be found in Table 1. In the IVW approach, Mendelian randomization analysis using 53 SNPs showed a significant causal relationship between CRP levels and ISSHL risk (OR = 1.23, 95% CI = 1.02–1.49, *P* = 0.03). These results remained broadly consistent in the supplementary weighted median analysis (OR = 1.45, 95% CI = 1.56–1.98, *P* = 0.02). Scatter and forest plots also showed a statistically significant association between genetic susceptibility to CRP levels and ISSHL (Figure 2). There was also no evidence of observed heterogeneity in effect estimates between variants (Cochrane's *Q* = 52.54, *P* = 0.45), and the funnel plot visualization is shown in Figure 3. The MR-PRESSO method also did not detect any abnormal IV. The results of the leave-one-out sensitivity analysis suggest that the association between CRP and ISSHL is not due to a single SNP (Figure 4). Importantly, MR-Egger regression revealed no evidence of directional pleiotropic effects in genetic variation (Intercept = 0.009, Se = 0.01, *P* = 0.29). This strongly suggests that our analysis is robust.

**Table 1:** MR analyses showing the associations of genetically Inflammatory markers with the Risk of ISSHL.

**MR approaches**	**SNPs**	**OR**	**(95% CI)**	**P-value**
**CRP**				
IVW	53	1.45	(1.05, 1.98)	0.03
Weighted median	53	1.23	(1.02, 1.49)	0.02
MR-Egger	53	1.10	(0.83, 1.46)	0.49
MR-PRESSO	53	/	/	0.36
**TNF-α**				
IVW	5	1.14	(0.88, 1.49)	0.30
Weighted median	5	1.19	(0.85, 1.66)	0.30
MR-Egger	5	1.07	(0.68, 1.70)	0.78
MR-PRESSO	5	/	/	0.52
**Fibrinogen**				
IVW	18	1.04	(0.88, 1.25)	0.59
Weighted median	18	1.60	(0.85, 1.31)	0.60
MR-Egger	18	1.40	(0.97, 2.04)	0.09
MR-PRESSO	18	/	/	0.11

### 2.7. Causal association of TNF-α and fibrinogen with ISSHL

**Figure 5:**
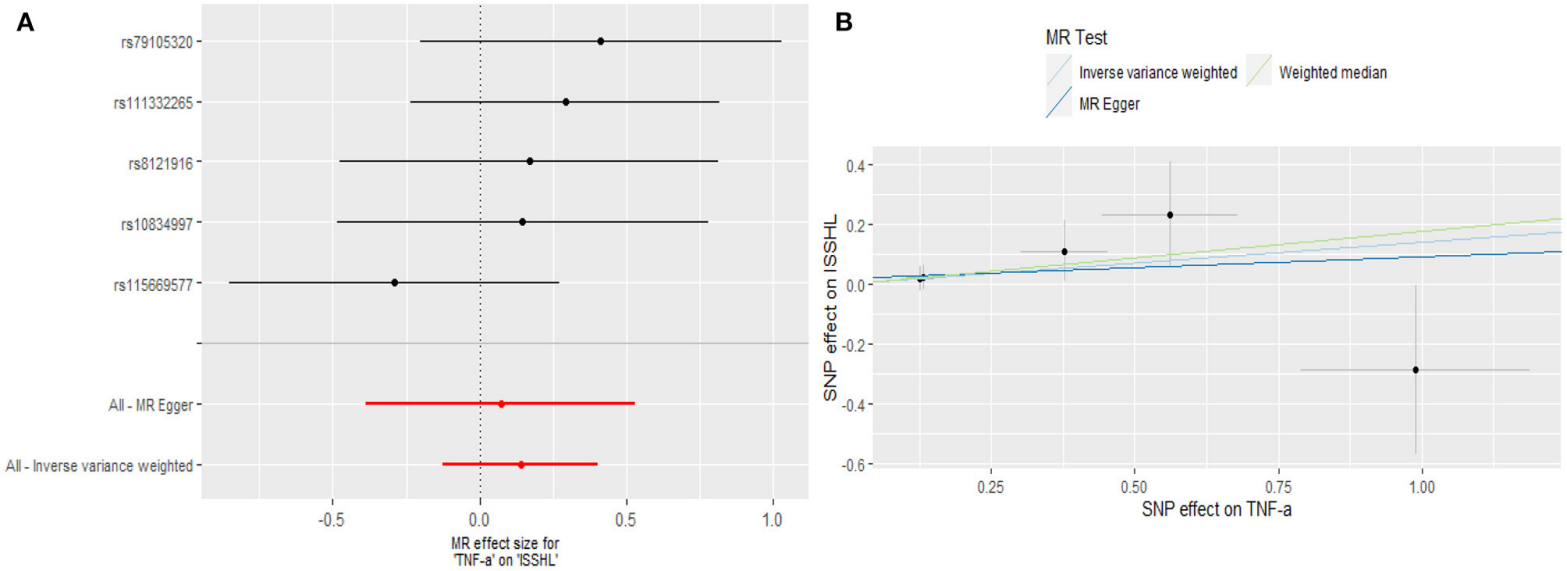
**(A)** Forest plot and **(B)** scatter plot of the potential effects of TNF-α associated SNPs on ISSHL.

**Figure 6:**
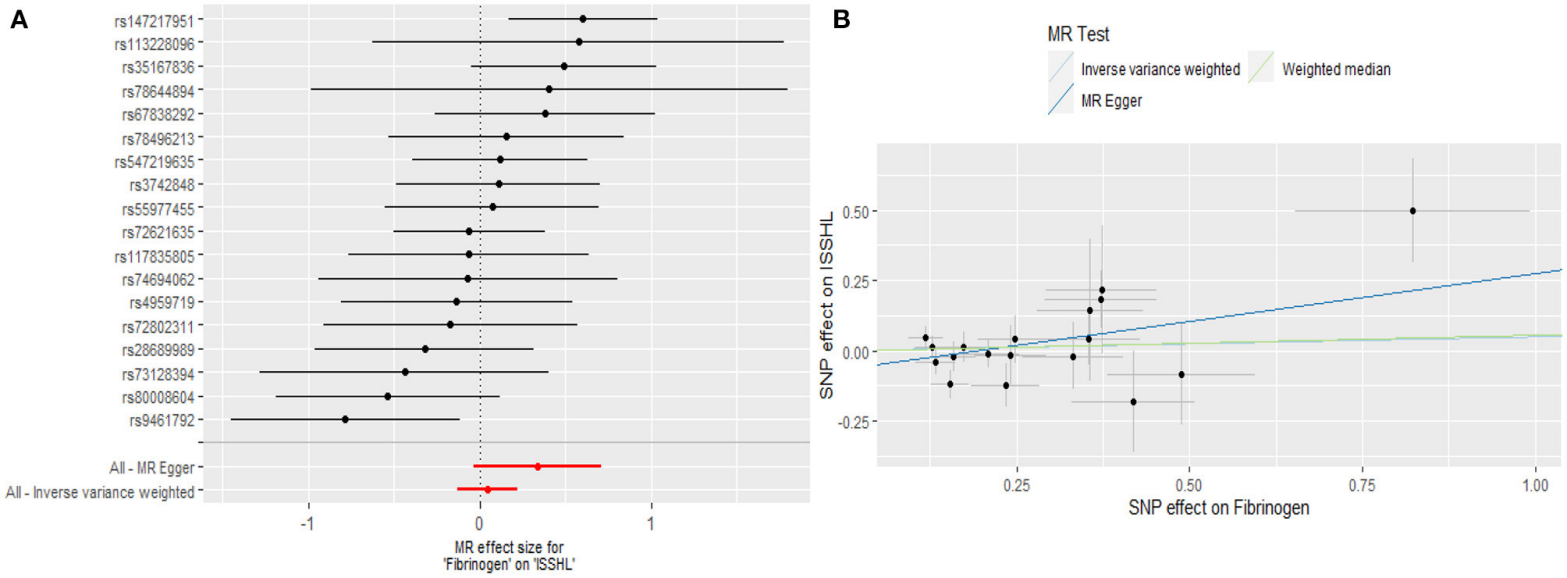
**(A)** Forest plot and **(B)** scatter plot of the potential effects of Fibrinogen associated SNPs on ISSHL.

Regarding TNF-α and fibrinogen, no evidence supported the hypothesis that they are associated with the ISSHL results of the main IVW methods. Furthermore, the weighted median, MR-PRESSO, and MR-Egger methods in sensitivity analyses support the above statement, as shown in Table 1. Forest and scatter plots also show MR estimates of the association between each TNF-α and fibrinogen-related SNP and the risk of ISSHL (Figures 5, 6). MR-Egger method showed that TNF-α (Intercept = 0.017, Se = 0.05, *P* = 0.74) and fibrinogen (Intercept = 0.069, Se = 0.15, *P* = 0.69) were not directionally pleiotropic with ISSHL. Cochrane Q-statistics for TNF-α (*P* = 0.50) and fibrinogen (*P* = 0.13) also showed no heterogeneity. None of the MR-PRESSO methods identified potentially aberrant SNPs. A leave-one-out analysis showed that a single SNP did not influence the estimates of TNF-α and fibrinogen with ISSHL (Figure 4).

## 3. Discussion

Based on large-scale GWAS data from the current European population, the two-sample MR study suggests a vital role in a positive association between CRP levels and the risk of ISSHL, while our results also indicate that TNF-α and fibrinogen are not significantly associated with the development of ISSHL. These results remain robust when validated by multiple sensitivity analyses, supporting our conclusions.

Chronic inflammation not only contributes to the development of the disease but also influences its prognosis. As the disease progresses, it increases the likelihood of microvascular damage and ischemia, promoting atherosclerosis (26). The cochlea is supplied by a single blood vessel (the labyrinthine artery), and the hair cells of the cochlea are susceptible to hypoxia, which can lead to cochlear dysfunction (27). CRP, a widely studied acute phase protein, may be involved in the pathogenesis of atherosclerosis by activating complement, stimulating monocyte chemotaxis, and inhibiting neutrophil chemotaxis *via* the classical pathway, and there is increasing evidence that it is a risk factor for atherosclerosis (28). TNF-α has been identified as a representative of one of the most important regulators of the immune system and can influence the activation of many intracellular signaling pathways through various routes, ultimately leading to cell survival, cell migration, apoptosis, and necrosis (29). In addition, a sudden decrease in blood flow in the vagus artery and thrombosis leading to microcirculation in the cochlea play an essential role in developing hearing loss (30). As an indicator of blood viscosity, fibrinogen is involved in various processes, such as platelet aggregation and activation of inflammation, suggesting a possible link between fibrinogen in serum and reduced blood flow in the cochlea (31). The results of this work are consistent with previous studies where a meta-analysis summarizing 13 years of English literature showed that CRP levels were higher in ISSHL patients than in the average population, while TNF-α results were heterogeneous (11). Another meta-analysis found no difference in fibrinogen concentration between control and SSNHL patients (10). However, in a recent study, CRP, TNF-α, and fibrinogen were shown to be associated with ISSHL outcomes to some extent (32). Another retrospective observational study associated fibrinogen with ISSHL risk (33). However, because most conventional studies to date have been observational or cohort studies, which are susceptible to potential confounders and causality reversals, as well as biases due to insufficient sample sizes, different disease diagnostic criteria, and diverse populations that limit interpretation of results, it is still not known whether these three inflammatory markers play a role in triggering the risk of ISHHL.

It is worth noting that this is the first two-sample MR study to investigate the causal relationship between CRP, TNF-α, fibrinogen, and ISSHL from a genetic perspective. The strength of this study is that the correlation between these three factors and ISSHL risk was analyzed using large-scale GWAS data from European populations, reducing the bias due to population stratification and making our statistical power very strong and convincing. A two-sample MR design was also used to provide causal evidence that CRP is a risk factor for ISSHL, while the association of fibrinogen and TNF-α with ISSHL was not significant, mainly eliminating the impact of limitations of conventional studies.

However, some limitations in this study need to be taken into account. First, the participants of GWAS involved in the study were limited to Europe with big data, and it remains highly questionable whether the same results can be extrapolated to other ethnic groups. Secondly, the lack of stratified data such as gender and age in the existing aggregated statistical sets prevents us from conducting a comprehensive and more granular analysis. If more detailed, publicly available stratification data were available in the future, this would allow further specification of the new MR analysis. Finally, the relatively small sample size of the GWAS for TNF-α and fibrinogen may lead to some bias in the results. Therefore, we should be cautious in interpreting the negative results of the effects of TNF-α and fibrinogen on the risk of ISSHL.

In conclusion, this MR study provided preliminary genetic evidence for a causal relationship between CRP levels and increased ISSHL risk but no proof of a causal relationship between TNF-α and fibrinogen and ISSHL. These findings may provide new ideas for understanding the relationship between inflammatory markers and ISSHL, and additional studies are urgently needed to validate our findings further.

## Data availability statement

The original contributions presented in the study are included in the article/Supplementary material, further inquiries can be directed to the corresponding author.

## Author contributions

TZ: study conception and design. MC and ZY: data analyses. TZ and ZX: draft preparation. SZ and HC: prepared figures. RL and ZZ: supervision of the study. All authors reviewed the manuscript. All authors contributed to the article and approved the submitted version.
